# Case Report: Vanishing bile duct syndrome in Hodgkin’s lymphoma: a case highlighting jaundice and lymphadenopathy as early clues

**DOI:** 10.3389/fimmu.2025.1561110

**Published:** 2025-05-21

**Authors:** Shiyu Ma, Dali Cai, Yuan Miao, Baocheng Deng, Xiaojing Yan, Ran Gao

**Affiliations:** ^1^ Department of Hematology, The First Hospital of China Medical University, Shenyang, Liaoning, China; ^2^ Department of Pathology, The First Hospital of China Medical University, Shenyang, Liaoning, China; ^3^ The Second Department of Infectious Diseases, The First Hospital of China Medical University, Shenyang, Liaoning, China

**Keywords:** jaundice, Hodgkin’s lymphoma, vanishing bile duct syndrome, liver biopsy, brentuximab vedotin (BV)

## Abstract

Vanishing bile duct syndrome is a specific pathologic process characterized by ductopenia and intrahepatic cholestasis, which may be a unique paraneoplastic syndrome of Hodgkin’s lymphoma with an unfavorable prognosis. We report a 34-year-old woman with acute jaundice and lymphadenopathy, which was subsequently confirmed to be Hodgkin’s lymphoma with concurrent vanishing bile duct syndrome based on a liver biopsy. The patient agreed to combination chemotherapy with brentuximab vedotin and achieved a complete response. Liver function recovered within 4 months. This article reviews the literature and provides insight for addressing similar clinical challenges.

## Case presentation

A 34-year-old female was admitted to the Department of Gastroenterology with rapidly progressive jaundice for 1 week and persistent lumbar pain for 1 month. She had pruritus, dizziness, fatigue, nausea, anorexia, and transient diarrhea for 2 weeks, as well as postpartum night sweats and weight loss over the preceding 6 months. She described dark urine and pale stools for 1–2 weeks. She denied fevers, dyspnea, or arthralgias. There was no personal or family history of hepatic disease and hematologic disorders. Prior to admission she was taking celecoxib (200 mg orally twice daily) and applying diclofenac diethylamine emulgel for 2 weeks to relieve the lumbar pain. Laboratory testing showed significant abnormalities in hepatic function, as follows: aspartate aminotransferase (AST), 87 U/L; alanine aminotransferase (ALT), 123 U/L; gamma-glutamyl transferase (GGT), 306 U/L; alkaline phosphatase (ALP), 997 U/L; total bilirubin, 146.2 μmol/L; direct bilirubin, 113.2 μmol/L; total cholesterol, 7.88 μmol/L; and lactate dehydrogenase (LDH), 525 U/L. During the admission, the ALT, GGT, ALP, and total bilirubin levels gradually increased to 10–20 times the upper limit of normal. A magnetic resonance cholangiopancreatography showed no evidence of biliary obstruction.

All the findings indicated intrahepatic cholestasis, but the cause was still unknown. The patient and her family denied a similar history. Laboratory examinations of rheumatism showed no apparent abnormality, except SSA IgG (95.9 U/mL). Considering the history of celecoxib use, a probable diagnosis of drug-induced liver damage was made.

**Figure 1:**
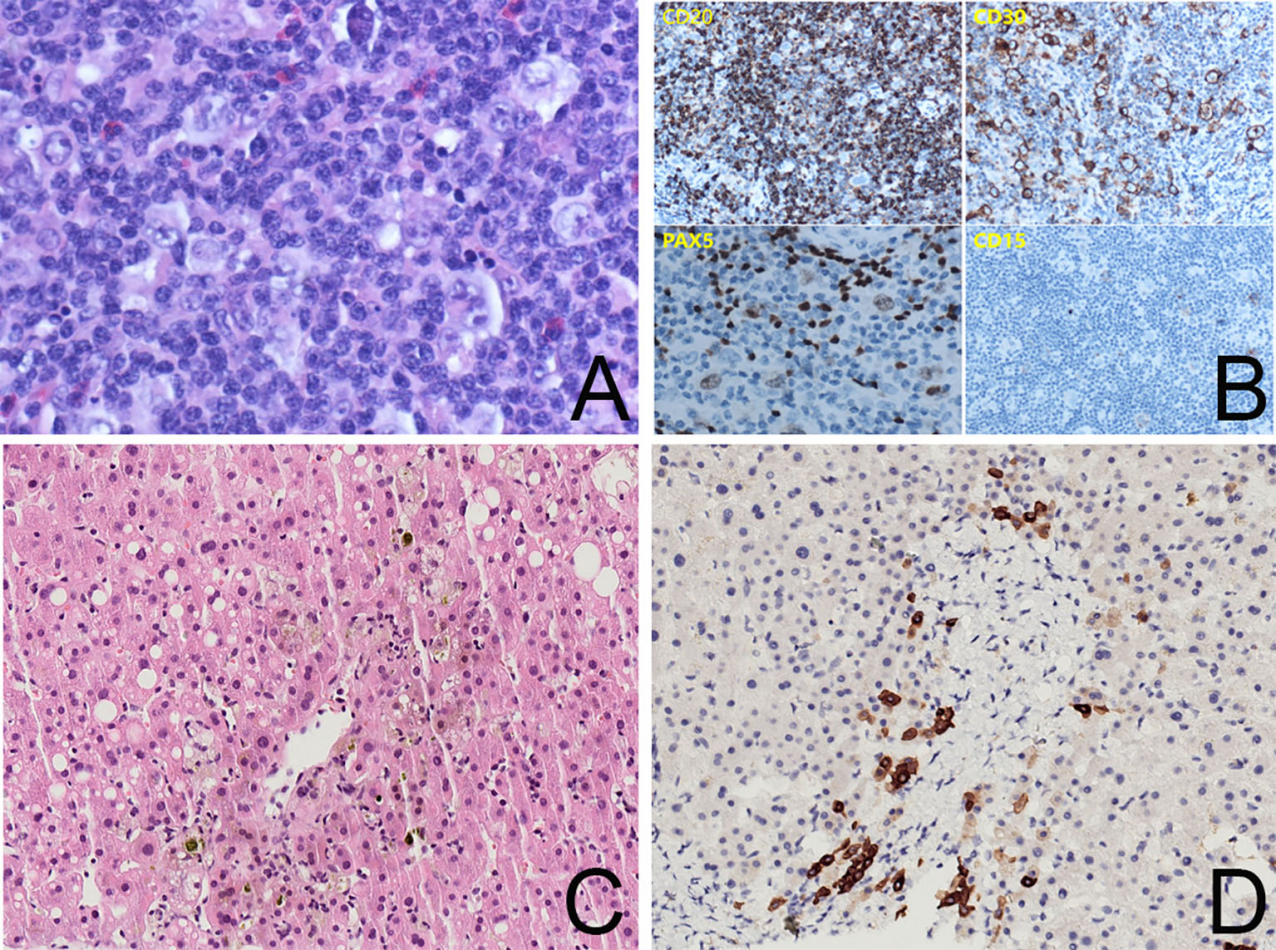
**(A)** Lymph node biopsy, ×400 magnification, showing R-S cells in a background of diffuse inflammation. **(B)** Immunohistochemistry staining for CD20, CD30, PAX5, and CD15 showing positive expressions in tumor cells. **(C)** Liver biopsy, ×200 magnification, HE immunohistochemistry stain, showing intrahepatic cholestasis around the central vein and macrovesicular steatosis in a few hepatocytes. The capillary bile ducts were blocked and dilated. There was no evidence of lymphoma. **(D)** Liver biopsy, ×200 magnification, CK7 immunohistochemistry stain showing hepatocytes with positive staining and loss of small bile ducts in the interstitium of the portal area. There was no sign of a bile ductular reaction.

Ursodeoxycholic acid (UDCA) and transmetil were used to relieve the jaundice. Furthermore, the physical examination revealed enlarged cervical lymph nodes (1–2 cm), thus neoplastic diseases were considered. Chest and abdominal computed tomography (CT) scans revealed generalized enlarged lymph nodes, splenomegaly, and bone changes in the T12 and L1 vertebrae. Positron emission tomography (PET)/CT was arranged to further assess pathologic changes in the left neck, mediastinum, retroperitoneum, liver, spleen, bone, and bone marrow. A left cervical lymph node biopsy was performed and the diagnosis of classical Hodgkin’s lymphoma (cHL) was established based on a background of diffuse inflammation and scattered large, atypical cells expressing CD15, CD30, and PAX5 (**Figures 1A, B**).

The diagnosis was confirmed to be stage IVB cHL. The jaundice was thought to be caused by liver involvement of the lymphoma. Indeed, jaundice occurs in 3%–13% of lymphoma patients with liver involvement (1) and is usually accompanied by elevated liver enzymes and/or bilirubin. Early intervention, including combination chemotherapy, has been shown to effectively eliminate the jaundice.

The patient received a 5-day course of dexamethasone at a dose of 10 mg followed by adriamycin, bleomycin sulfate, vinblastine sulfate, and dacarbazine (ABVD). The total bilirubin level decreased significantly the next day (358.5 to 295.1 μmol/L), then increased to 395.4 μmol/L.

The persistent, worsening jaundice led us to reconsider another possible etiology. Although the PET/CT scan suggested liver involvement, the standardized uptake value was lower than lymph nodes and other involved areas, suggesting that there would be an inadequate cause for severe intrahepatic cholestasis.

Vanishing bile duct syndrome (VBDS) was included in the differential diagnosis after a multi-disciplinary treatment discussion. The patient accepted a transjugular liver biopsy to confirm the diagnosis. The hepatic histopathologic evaluation revealed absence of bile ducts in 7 of the 11 portal areas and periportal hepatocytes with cholate stasis (**Figures 1C, D**). There was no evidence of lymphoma.

VBDS is a specific pathologic process that is characterized by ductopenia and intrahepatic cholestasis (2). The diagnosis is difficult to establish without performing a liver biopsy. There are many causes of VBDS, including drugs, infections, tumors, and immunologic disorders. HL has been reported to be the most common malignancy associated with VBDS. VBDS-associated HL has a poor prognosis because chemotherapy is delayed and often leads to hepatic failure or sepsis (3).

According to the recent literature, it is relatively safe for most patients to accept a modified chemotherapy regimen with low liver toxicity or molecularly targeted drugs. Brentuximab vedotin (BV) is a new and potentially effective treatment for VBDS-associated HL by avoiding hepatic damage caused by chemical drugs, as well as VBDS (4, 5).

**Figure 2:**
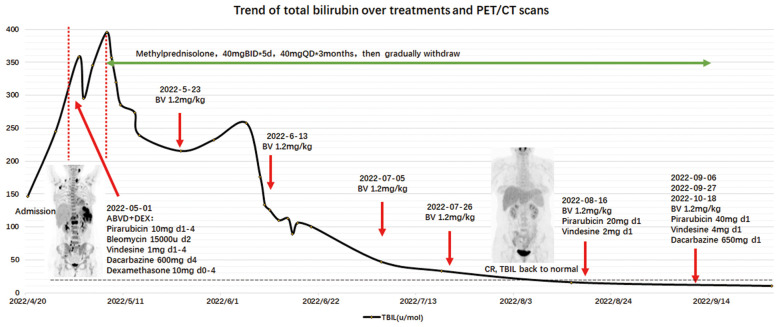
Trend of total bilirubin level over treatments and PET/CT scans.

After one-half of the ABVD regimen had been administered, the patient expressed concerns about combination chemotherapy and opted for treatment with BV with methylprednisolone (40 mg daily) and ursodeoxycholic acid (UDCA) to reduce liver damage. Given four cycles of BV (1.2 mg/kg), the bilirubin level returned to normal (18 μmol/L) and the PET/CT scan showed a complete metabolic response (CMR), suggesting that VBDS was reversed with remission of the lymphoma. Thereafter, combination chemotherapy was added (BV+AV*1, BV+AVD*3). A subsequent PET scan showed pelvic wall and mesentery lymph node progression (Deauville score = 4), so five cycles of anti-PD-1+ GVD were administered. Because the PET/CT scan showed slight uptake in the left cervical lymph nodes, the patient accepted a second biopsy to determine if regional radiation therapy should be continued. The biopsy results revealed lymphoid hyperplasia and no evidence of a lymphoma. The patient was prepared for an autologous hematopoietic stem cell transplantation with anti-PD-1 therapy as maintenance treatment. Liver function remained within normal limits until October 2023 except GGT (81 U/L) and ALP (314 U/L), as shown in **Figure 2**.

## Discussion

VBDS associated with lymphoma was first described in 1993 (2). VBDS is mostly described as a unique paraneoplastic syndrome of Hodgkin’s lymphoma that has an unfavorable prognosis. The pathogenesis of VBDS associated with lymphoma has not been established. Some studies speculate that VBDS is relevant to the toxic cytokines released by lymphoma cells (2, 6, 7). T lymphocytes may respond to the cytokines and therefore cause adhesion and cytotoxicity to biliary epithelial cells (8, 9). This process possibly predates any other clinical signs of lymphoma, so it is difficult to identify the real illness.

**Figure 3:**
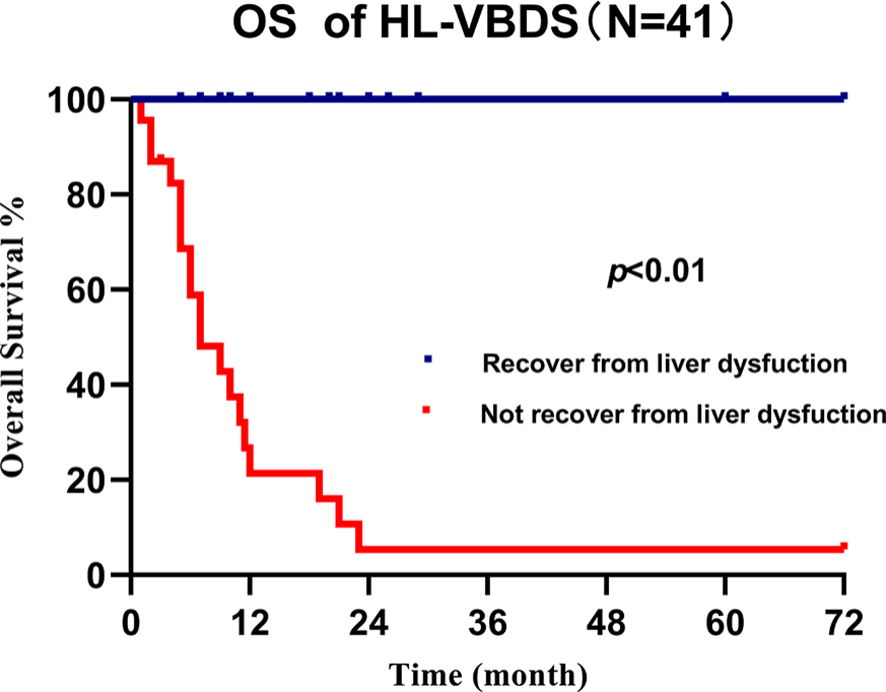
Overall survival of cHL-associated VBDS grouped by recovery from liver dysfunction, which showed that recovery of liver function benefits survival.

We retrospectively analyzed 25 cases of lymphoma accompanied by VBDS reported in the last 10 years (4, 10–32) and came to the conclusion that due to the confidence in treating the primary disease, early recovery of liver function (represented by the decrease in bilirubin level) is the main factor that predicts the survival of patients with VBDS-associated lymphoma (**Figure 3**, *P* < 0.01). However, long-term survival rate of HL with VBDS has not been adequately addressed in the literature.

This case suggests that VBDS should be considered as a specific cause of jaundice associated with lymphoma and not only the tumor involvement. When the jaundice fails to recover quickly after systemic treatment of the primary malignancy, a liver biopsy should be performed to determine the possible cause as soon as possible.

Because the diagnosis of VBDS is confirmed by the loss of > 50% of interlobular bile ducts in an adequate biopsy specimen (containing ≥ 10 portal tracts), nearly all patients diagnosed with VBDS undergo liver biopsy. However, due to safety concerns, patients might decline a biopsy. Since some cases of intrahepatic cholestasis may only be revealed as VBDS based on liver biopsy results, the true prevalence of VBDS is very likely underestimated (2). As previously reported, ultrasound-guided percutaneous liver biopsy (PLB) is the most reliable and common method for the definitive diagnosis of VBDS in addition to liver autopsy. In our case, given the patient’s elevated bleeding risk and hyperbilirubinemia, CT-guided transjugular liver biopsy (TJLB) seemed to be a better way to reduce liver capsule damage. Although TJLB is not as accurate as PLB in patients with precisely located liver lesions, TJLB is considered more suitable for patients with diffuse liver injury.

In addition, there are many causes of VBDS, as in the current case. Drug and tumor factors are mixed, therefore it may be difficult to distinguish the chief cause of VBDS. Although coxib-induced liver injury is regarded as an uncommon event, it is undeniable that celecoxib may aggravate the side effects of chemotherapy on the liver (33). We demonstrated that the accumulation of cholate stasis in periportal hepatocytes with the absence of small bile ducts was gradually forming based on the liver pathologic findings, which was greater than what would be expected after 2 weeks of celecoxib use. Therefore, the pathologic diagnosis of this case was more appropriately referred to as VBDS caused by HL with drug-induced liver injury, rather than VBDS caused by drug-induced liver injury.

To our knowledge, VBDS may occur before, during, or after the onset of lymphoma and sometimes reappears when the lymphoma relapses (34). Assuming that the bile duct epithelial cells could recover from damage in some optimally treated patients, explains why most patients with HL-VBDS are tolerant to chemotherapy. The clinical efficacy was not significantly affected in the mid-term evaluation.

The chemotherapy interval is prolonged and the intensity is decreased due to VBDS, which may ultimately decrease patient survival. Our patient was shown to progress after the seventh cycle evaluation, which was a sign that single-agent BV was less sufficient for the treatment of stage IVB HL, even though single-agent BV had an acceptable safety profile and rapidly normalized the bilirubin level within 4 months. Combination chemotherapy might have been added as early as possible. A similar patient diagnosed with stage III HL was treated with BV, cyclophosphamide, prednisolone, and procarbazine. She also achieved a CMR in the mid-term evaluation with the total bilirubin level back to normal in approximately 5 months but relapsed 3 months after drug withdrawal. She finally achieved a PMR after 8 cycles of the ABVD regimen (17). Our treatment with single-agent BV was associated with a better safety profile, earlier recovery of liver function, and an encouraging complete remission after treatments.

Because liver failure is the leading cause of death reported previously in the literature, we should be aware of the benefits of single-agent BV in VBDS-associated lymphoma. Considering that three of four BV-involved patients achieving a CR, BV showed good survival benefits. A rapid recovery from liver lesions provides patients with an opportunity to undergo more adequate chemotherapy regimens and avoids adverse outcomes, such as severe coagulopathy or sepsis.

This case improves the clinical understanding of cHL accompanied by VBDS so that early detection with sufficient expectation of the disease episode can be made. A higher priority of BV in cHL with VBDS needs to be considered and further studies are required to determine the cause of cHL-VBDS. By summarizing the clinical features and adjusting the treatment of cHL-VBDS, we can avoid further damage of the organs and maximize the benefits.

## Patient Consent Statement

Informed consent was documented by means of a written, signed, and dated informed consent form from the patient.

“We agree to publish this case report and extend heartfelt gratitude to our medical team. By prioritizing patient-centered care and scientifically optimizing the treatment plan for severe liver dysfunction, they have given the patient a new lease on life.”

## Data Availability

The original contributions presented in the study are included in the article/**Supplementary Material**. Further inquiries can be directed to the corresponding authors.
