# Supplementary material for: Case Report: Vanishing bile duct syndrome in Hodgkin’s lymphoma: a case highlighting jaundice and lymphadenopathy as early clues
Source: Front Immunol. 2025 May 21;16:1561110. doi: 10.3389/fimmu.2025.1561110 (PMC12133851; doi:10.3389/fimmu.2025.1561110)
Supplement: Supplementary file 1 [file DataSheet1.pdf]

CARE
case report guidelines

# CARE Checklist of information to include when writing a case report

(cc) BY-NC-ND

| Topic | Item | Checklist item description | Reported on Line |
|---|---|---|---|
| **Title** | 1 | The diagnosis or intervention of primary focus followed by the words "case report" | L60-70 |
| **Key Words** | 2 | 2 to 5 key words that identify diagnoses or interventions in this case report, including "case report" | L89-91 |
| **Abstract (no references)** | 3a | Introduction: What is unique about this case and what does it add to the scientific literature? | L77-79 |
| | 3b | Main symptoms and/or important clinical findings | L79-84 |
| | 3c | The main diagnoses, therapeutic interventions, and outcomes | L82-84 |
| | 3d | Conclusion—What is the main "take-away" lesson(s) from this case? | L82-83 |
| **Introduction** | 4 | One or two paragraphs summarizing why this case is unique (**may include references**) | L278-295 |
| **Patient Information** | 5a | De-identified patient specific information. | L98-99 |
| | 5b | Primary concerns and symptoms of the patient. | L99-102 |
| | 5c | Medical, family, and psycho-social history including relevant genetic information | L102-103 |
| | 5d | Relevant past interventions with outcomes | L116-117 |
| **Clinical Findings** | 6 | Describe significant physical examination (PE) and important clinical findings. | L99-109,L121-132 |
| **Timeline** | 7 | Historical and current information from this episode of care organized as a timeline | Figure 2 |
| **Diagnostic Assessment** | 8a | Diagnostic testing (such as PE, laboratory testing, imaging, surveys). | Figure 1 |
| | 8b | Diagnostic challenges (such as access to testing, financial, or cultural) | L302-358 |
| | 8c | Diagnosis (including other diagnoses considered) | L359-371 |
| | 8d | Prognosis (such as staging in oncology) where applicable | L288-295 |
| **Therapeutic Intervention** | 9a | Types of therapeutic intervention (such as pharmacologic, surgical, preventive, self-care) | L233-252 |
| | 9b | Administration of therapeutic intervention (such as dosage, strength, duration) | L233-250 |
| | 9c | Changes in therapeutic intervention (with rationale) | L379-394 |
| **Follow-up and Outcomes** | 10a | Clinician and patient-assessed outcomes (if available) | L238-252 |
| | 10b | Important follow-up diagnostic and other test results | L249-252 |
| | 10c | Intervention adherence and tolerability (How was this assessed?) | L233-236, L244-250 |
| | 10d | Adverse and unanticipated events | L379-394 |
| **Discussion** | 11a | A scientific discussion of the strengths AND limitations associated with this case report | L352-358 |
| | 11b | Discussion of the relevant medical literature **with references**. | L386-394 |
| | 11c | The scientific rationale for any conclusions (including assessment of possible causes) | L288-L295 |
| | 11d | The primary "take-away" lessons of this case report (without references) in a one paragraph conclusion | L395-402 |
| **Patient Perspective** | 12 | The patient should share their perspective in one to two paragraphs on the treatment(s) they received | L414-419 |
| **Informed Consent** | 13 | Did the patient give informed consent? Please provide if requested | Yes ☑ No ☐ |
